# Comparison of reduction of edema after rest and after muscle exercises in treatment of chronic venous insufficiency

**DOI:** 10.1186/1755-7682-2-18

**Published:** 2009-07-14

**Authors:** Belczak Cleusa Ema Quilici, Cavalheri Gildo, Jose Maria Pereira de Godoy, Belczak Sergio Quilici, Caffaro Roberto Augusto

**Affiliations:** 1 Vascular Surgery Service, Santa Casa Medicine School, São Paulo FCMSCSP-Brazil; 2 Maringá Vascular Center, Brazil; 3 Department of Cardiology and Cardiovascular Surgery, São Jose do Rio Preto Medicine School -FAMERP-Brazil; 4 Vascular Surgery Service, University of São Paulo, USP-Brazil

## Abstract

**Aim:**

The aim of this work was to compare the reduction in edema obtained in the conservative treatment of phlebopathies after resting and after performing a muscle exercise program in the Trendelenburg position.

**Methods:**

Twenty-eight limbs of 24 patients with venous edema of distinct etiologies and classified as between C3 and C5 using CEAP classification. Volumetric evaluation by water displacement was carried out before and after resting in the Trendelenburg position and after performing programmed muscle exercises 24 hours later under identical conditions of time, position and temperature. For the statistical analysis the paired t-test was used with an alpha error of 5% being considered acceptable.

**Results:**

The average total volume of the lower limbs was 3,967.46 mL. The mean reduction in edema obtained after resting was 92.9 mL, and after exercises it was 135.4 mL, giving a statistically significant difference (p-value = 0.0007).

**Conclusion:**

In conclusion, exercises are more efficient to reduce the edema of lower limbs than resting in the Trendelenburg position.

## Introduction

It is well known that edema, as a consequence of prolonged venous stasis of the lower limbs, causes and preserves trophic complications that are established during the evolution of this ailment. High walking venous pressures have been considered an important sign of phlebopathies, and thus, measures that aim not only to reduce walking hypertension but also combat the edema are recommended in chronic venous insufficiency (CVI) to control and/or impede the morbid evolution of the disease, whether superficial, deep or perforator [1-3].

Recent studies of the venous physiology have suggested that, to overcome the detrimental effects of gravitational force when standing, the venous system of lower limbs provides a hemocinetic apparatus constituting a lateral injection pumps which are composed of suction impulse pumps (SIP) or muscle-venous-articular pumps (MVAP). Usually, apart from prescribing medications and compressive therapy, other measures are recommended including resting in the Trendelenburg position for long periods of time and, more recently, as an integral part of the conservative clinical treatment, muscle exercises that activate the SIP and cause a reduction in the edema of the affected limb [4-9].

They produced a consensus document for the classification and grading of chronic venous disease, the CEAP classification is classified according to clinical signs (C), cause (E), anatomic distribution (A), and pathophysiologic condition (P) [10].

The aim of this work was to compare the reduction in edema obtained in the conservative treatment of phlebopathies after resting and after performing a muscle exercise program in the Trendelenburg position.

## Materials and methods

Twenty-eight limbs of 24 patients with CVI of distinct etiologies were selected for this study. The limbs were classified as C3 (8 limbs), C4 (9 limbs) and C5 (11 limbs) according to the clinical, epidemiological, anatomical and physiopathologic characteristics (CEAP) [11]. Of these patients, 3 were male and 21 female with ages that varied between 36 and 74 years old and a mean of 53.08 years. Fourteen right and 14 left lower limbs were analyzed.

All the limbs were submitted to a Doppler color imaging examination which confirmed the patency of both the superficial and deep venous systems and the existence of superficial and/or deep and/or oblique vertical truncal reflux due to incompetent perforators. All patients presented with flexibility of the ankle of more than 40° as measured by a goniometry technique.

Individuals with arterial hypertension and those who were undergoing hormonal replacement therapy were excluded from the study. Moreover, patients suffering from illnesses which limited the mobility of the talocrural joint for other reasons such as orthopedic or rheumatic diseases and patients with edema originating from traumatic, allergic, ischemic, renal, thyroidal, hepatic or cardiological causes were also excluded.

The patients, after a normal working day without using any compressive or medical measure that would interfere in the formation of edema of the lower limbs, arrived at the clinic for treatment at about 5:45 p.m. where they sat for around 15 minutes before starting the study protocol. On the first day they remained resting in the 30° Trendelenburg position at room temperature for about 30 minutes. On the second day, under exactly the same conditions in the Trendelenburg position, they performed an average of 10 plantar dorsiflexion exercises with a 4 kg load per minute for the same length of time as the resting on the previous day. Volumetry using water displacement was performed before and after each phase of the experiment [10].

The study was approved by the Ethics Committee of a local university.

For statistical analysis the paired student t-test was utilized with an alpha error of 5% being considered acceptable.

## Results

Table 1 shows the variables assessed and the results obtained. The mean reduction of edema during the exercises was 135.4 mL and with resting it was 92.9 mL (p-value = 0.0007).

**Table 1:** Individual variables and results before and after resting and exercises.

**N**	**AGE**	**SIDE**	**GENDER**	**CEAP**	**Total Vol.**	**Volume Post-rest**	**Volume Post-exercise**
01-	60	R	F	C3	3570	-120	-1–145
02-	47	L	F	C5	4845	-25	-90
03-	66	L	F	C5	3390	-40	---125
04-	40	L	F	C3	3040	-50	-88
05-	50	R	F	C3	3970	-80	-110
06-	45	L	F	C3	4480	-100	-140
07-	56	R	F	C3	3902	-65	-230
08-	74	L	F	C4	3275	-40	-69
09-	49	L	F	C4	4060	-280	-216
10-	53	R	M	C5	3410	-113	-120
11-	57	R	F	C4	2990	-160	-190
12-	45	L	F	C4	4310	-65	-95
13-	50	R	F	C3	4121	-20	-132
14-	69	L	F	C5	4200	-60	-90
15-	61	R	F	C5	4775	-65	-115
16-	46	L	F	C4	4910	-100	-110
17-	72	R	M	C5	5420	-164	-245
18-	39	R	F	C5	3431	-66	-278
19-	63	L	F	C5	4960	-85	-140
20-	63	R	F	C4	3610	-30	-90
21-	-	L	F	C3	3270	-40	-90
22-	38	R	F	C5	3360	-90	-30
23-	-	L	F	C4	3220	-100	-95
24-	60	R	F	C4	4700	-80	-90
25-	-	L	F	C5	4490	-80	-110
26-	54	R	F	C3	4700	-70	-20
27-	-	L	F	C4	4490	-395	-420
28-	36	R	M	C5	4775	-20	-120

## Discussion

In this study, individuals with good ankle motion were chosen despite the fact that they suffered from CVI, as the degree of flexibility of this joint is essential for the efficient functioning of the calf muscle pump (CMP) as the traction of the Achilles tendon depends on the contraction of the sural triceps.

Forty-five minutes was chosen for the time of the test because of a NHI Consensus Statement published by Leon in 1997 about physical activities and cardiovascular health which determined that a minimum of 30 minutes is required for the benefits of exercising to be seen [12]. During muscle exercises, contraction increases the external pressure on the veins and propels the blood back to the heart, reducing the hydrostatic pressure gradient that results in an edema [13].

On the other hand, it has been proven that raising the lower limbs increases the velocity of the blood by about 200%. Resting in the Trendelenburg position effectively corrects venous edema and reduces the risk of deep venous thrombosis (DVT). This position recommended for resting was utilized in the current study.

According to Ramelet et al. three simple 20 to 30-minute resting periods daily with the legs at an angle of 30° are sufficient to reduce the edema that affects the elderly [14].

Despite of the care in excluding patients with obstructive problems, one of the limitations of our study is not having excluded patients with post-thrombotic syndrome (PTS), those with only superficial systemic insufficiency.

Conservative therapy for many patients with severe CVI, especially those who present with PTS, is very limited [15].

Based on current knowledge of venous physiology, it is possible to affirm that movement is essential for an adaptation of men to their life style [14]. During walking, particularly with compression of the venous collectors of the plantar and calf muscles, as occurs with plantar bending and stretching, the MVAP and SIP are activated, increasing venous return and principally, re-absorption of fluids, thereby reducing the edema.

Edema of the lower limbs can be measured by the dislocation of water (plethysmography in the ancient Greek manner) [16] or by indirect measurements which calculate the volume from the surface area of the limb. Considered by Perrin and Guex as the gold standard for this evaluation [17], volumetry by the displacement of water has proved to be a sensible and reliable method [18]. Repeated measurements of limbs submitted to different types of therapy have been very useful to assess the benefits obtained by each type of treatment [19].

Additionally, it has been demonstrated that old age also interferes in the talocrural mobility and thus, an intermediate age range was chosen for the participants in this study [20].

Many studies, such as the ones by Fleming et al. [21], MacKinnon [22] and MacNally et al. [23], demonstrated that exercises that mobilize the ankle significantly increase the venous return of the lower limbs.

The study of Kahn et al. in 2005 showed that the symptoms related to venous hypertension are worse in patients with PTS during walking or standing [15]; these authors stated that PTS limits exercising in the upright position and that these exercises aggravate the symptoms and signs of acute venous insufficiency. They observed that although patients with PTS have a similar heart performance during exercising on a treadmill, the time of exercising is generally reduced probably due to the increase in volume and the flexibility of the ankle.

In our study, there were no cases of obstructive syndrome, only the presence of reflux and as the exercises were performed in the Trendelenburg position, the gravitational force was eliminated, thereby creating a pressure gradient favorable for drainage (similar to resting). Probably this is the main reason by that, in contrast other studies performed in the standing position; we did not see an increase in the volume of the limbs in any of our cases. This data encouraged us to compare, as initially proposed, without other interfering factors.

Comparatively analyzing the data from this study, we can state that the exercises, apart from stimulating and improving the activity of the SIP and MVAP of the lower limbs, facilitated the venous return as has been demonstrated in other previous publications [9,24,25], causing a greater reduction in the edema than resting alone. This observation leads us to believe that adopting muscle exercise programs in the decubitus position with the limbs raised may be a clinical coadjuvant therapeutic measure useful in the treatment of CVI that can not be surgically corrected.

The perspectives of exercises associated with contention mechanisms or in isolation for the treatment of venous and lymphatic diseases depend on establishing the best method of utilization and this approach can only be defined through research. However, this manner of approaching venous diseases is complementary to treatment, specifically when improvements in the joint mobility and the functioning of the calf pump are required. General precautions related to exercising must be taken assessing all the aspects that may interfere including cardiac resistance and other comorbidities [26]. We believe that these activities will not only give physical development to patients but will also improve the well being as has been demonstrated by other activities [27,28]. The aging of the population leads to an increase in the prevalence of chronic diseases such as venous insufficiency and these patients may be indicated for exercises such as these, however much care must be taken with the elderly[29,30].

## Conclusion

In conclusion, active exercises in the Trendelenburg position were more efficacious than resting to reduce the edema of lower limbs.

## Competing interests

The authors declare that they have no competing interests.

## Authors' contributions

CEQB, GCJ, JMPG, SQB and RAC all participated in all phases of the study (design, collects of data, analyze statistics and conclusion of the study). All authors read and approved the final manuscript.

## References

[B1] Belczak Neto J, Belczak CEQ, Thomaz JB, Belczak CEQ (2006). Reabilitação cinesiofisiátrica do flebopata crônico. Tratado de Flebologia e Linfologia.

[B2] Dormandy JA (1997). Microcirculation and Venous leg Ulcers. Phlebolymphology.

[B3] Pereira de Godoy JM, Braile DM, de Fatima Guerreiro Godoy M (2008). Lymph drainage in patients with joint immobility due to chronic ulcerated lesions. Phlebology.

[B4] Godoy JMP, Godoy MFG (2004). Manual lymph drainage: a new concept. J Vasc Br March.

[B5] Artíbale MES, Godoy JMP, Godoy MFG, Braile DM (2005). A new option for compression in the treatment of lymphedema in children. J Vasc Br.

[B6] Brizzio EO, Mancini S (2001). Le pompe Impulse-Aspirative degli arti inferiori. Libro. Tratatto di Flebologia e Linfologia.

[B7] Brizzio EO (1988). Propellent Suction Pumps of the Lower Limbs. Angiologia.

[B8] Cavalheri G, de Godoy JM, Belczak CE (2008). Correlation of haemodynamics and ankle mobility with clinical classes of clinical, aetiological, anatomical and pathological classification in venous disease. Phlebology.

[B9] Belczak CEQ, Godoy JMP, Seidel AC, Silva JA, Cavalheri Junior G, Belczak SQ (2004). Influência da atividade diária na volumetria dos membros inferiores medida por perimetria e pela pletismografia de água. J Vas Br.

[B10] Eklof B (2006). CEAP classification and implications for investigations. Acta Chir Belg.

[B11] Partsch H, Thomaz JB, Belczak CEQ (2006). Classificação da Insuficiência Venosa Crônica- CEAP. Tratado de Flebologia e Linfologia.

[B12] Nilsson S, Bjerknes Haugen G (1981). Volumetry in the evaluation of swelling and foot. J Oslo City Hosp.

[B13] Belczak CEQ, Belczak Neto J (2001). Importância da ativação das bombas impulso-aspirativas no tratamento da hipertensão venosa crônica. Rev Bras Fleb e Linf.

[B14] Perrin M, Guex JJ (2000). Edema and Leg Volume: Methods of Assessment. Angiology.

[B15] Nigg BM, Frederik V, Allinger TL, Ronsky JR, Engsberg JR (1992). Range of motion of the foot as a function of age. Foot & Ankle.

[B16] Briele HA, Schneebaum S, Barnicle M, Briele C (1989). Method of measurement for volume of an extremity. Surg Gynecol Obstet.

[B17] Leon AS, NHI consensus statement (1997). Physical activity and cardiovascular health: a national consensus.

[B18] Fleming P, Fitzgerald P, Devitt A, Rice J, Murray P (2000). The effect of the position of the limb on venous impulse foot pumps. Journal of Bone and Joint Surgery.

[B19] Kahn SR, Azoulay L, Hirsch A (2003). Acute effects of exercise in patients with previous deep venous thrombosis. Chest.

[B20] Mackinnon JL (1983). Study of Doppler ultrasonic peripheral vascular assessments performed by physical therapists. Physical Therapy.

[B21] McNally MA, Cooke EA, Mollan RA (1997). The effect of active movement of the foot on venous blood flow after total hip replacement. J Bone Joint Surg Am.

[B22] Stick C, Grau II, Witzleb E (1989). On the edema-preventing effect of the calf muscle pump. Eur J Appl Physiol Occup Physiol.

[B23] Ramelet AA, Kern P, Perrin M, Ramelet AA, Kern P, Perrin M (2004). Lifestyle, physical therapy and prevention. Varicose veins and telangiectasias.

[B24] Belczak CEQ, Belczak Neto J, Godoy JMP, Belczak CEQ, Godoy MFG (2005). Reabilitação cinesioterápica da bomba muscular da panturrilha na insuficiência venosa crônica. Reabilitação Linfovenosa.

[B25] Yang GD, Vandongen YK, Stacey MC (1997). Variability and reliability of air plethysmographic measurements for the evaluation of chronic venous diseases. J Vasc Surg.

[B26] Quist M, Rorth M, Zacho M, Andersen C, Moeller T, Midtgaard J, Adamsen L (2006). High-intensity resistance and cardiovascular training improve physical capacity in cancer patients undergoing chemotherapy. Scand J Med Sci Sports.

[B27] Sjogren T, Nissinen KJ, Jarvenpaa SK, Ojanen MT, Vanharanta H, Malkia EA (2006). Effects of a physical exercise intervention on subjective physical well-being, psychosocial functioning and general well-being among office workers: a cluster randomized-controlled cross-over design. Scand J Med Sci Sports.

[B28] Jawień A, Szewczyk M, Kornatowska K, Mościcka P, Cierzniakowska K, Cwajda J, Polak A, Grzeszak I (2006). Functional and biopsychosocial restrictions among patients with a venous ulcer. Arch Med Sci.

[B29] Christensen U, Stovring N, Schultz-Larsen K, Schroll M, Avlund K (2006). Functional ability at age 75: is there an impact of physical inactivity from middle age to early old age?. Scand J Med Sci Sports.

[B30] Pereira De Godoy JM, Miquelin D, Braile DM, Menezes Da Silva A (2009). Association of deep venous thrombosis with neoplasms in different age groups. Int Angiol.

